# Social Integration and Community Health Participation of Elderly Men in Peri-Urban Ecuador

**DOI:** 10.5334/aogh.3020

**Published:** 2020-10-26

**Authors:** John DiBello, Luke Murphy, Iván Palacios

**Affiliations:** 1 Program in Global Public Health and the Common Good, Boston College, Chestnut Hill, MA, US; 2 Global Health Program, Universidad San Francisco de Quito Medical School, Quito, EC

## Abstract

**Background:**

Social integration is an essential element to the maintenance of health and well-being in elderly populations. In the Cumbaya Valley of Quito, Ecuador, community health clinics sponsor social clubs for specific populations to address this important aspect of health. Men, who tend to be less socially integrated than women, are largely absent from these programs.

**Objective:**

This paper investigates the quality and extent of men’s social integration in the Cumbaya Valley of Quito to understand why men are less likely to attend the community health center clubs and to develop ideas for increasing male participation, which may differ from current methods.

**Methods:**

A composite survey was used to interview 100 men over the age of 40 to collect data on their social health and information regarding their interaction with community health center clubs and other local social groups.

**Findings:**

Social integration scores were varied, with some men having high social scores and others having low scores. Men generally had greater access to affectionate and tangible support but lower access to emotional support and positive social interaction. Men spend far more social time with their families and much less with friends and neighbors. Regression analysis revealed that social scores have a relationship with age and education. Qualitative results suggest gendered expectations of men in the community have negatively impacted their willingness to engage in community health groups. Participants also provided suggestions, including specific sports, gardening, and meal distribution, to promote male participation.

**Conclusion:**

There is a strong need to increase services, strategies, and programs that address the lack of social integration experienced by men. This paper presents the particular role community clinics can play in increasing the social well-being of its male patients.

## Introduction

Social integration is an essential element in the health of older individuals, demonstrated through the extensively researched “Village Effect” [1]. Robust socialization in a community correlates with longer and healthier lives. Additionally, “people with the most integrated social lives— meaning those who have overlapping relationships among friends, family, sports and other recreational or religious pursuits— have the best prognoses” for heart attacks, strokes, HIV/AIDS, and cancer, among other conditions [1]. Case studies can be drawn from particular geographical pockets where life expectancy and happiness ratings are uncharacteristically high [2]. A frequently cited example is Villagrande in Sardinia, Italy, where “ten times as many men…live past the age of one hundred as men who live elsewhere” [1]. In this town, men have very active social and community engagement. Active participation in a diversity of social activities acts as a protective measure from both communicable and non-communicable diseases. In an assessment of nearly seven thousand citizens from Finland, “one of the most powerful predictors of loneliness was living alone; the lonely folks…were 31 percent more likely to have died…than people who felt intimately connected” [3]. A supportive social network facilitates better health behaviors, including smoking cessation and increased exercise [4]. Moreover, “socially isolated seniors are more at risk of negative health behaviors,” with social isolation linked to a greater risk of longer and more frequent hospitalizations as well as lower cognitive functioning [4,5]. A systematic review of 34 articles relates both music and physical activity programs with increased cognitive performance in older adults [6]. Exercise, aerobic in particular, is associated with an increase in hippocampal volume, improving cognition and protecting against memory loss in late adulthood [7]. The positive implications of social integration and physical activities in the community are broadly-scoped.

While patriarchy privileges men in most social and economic contexts, there are persistent disparities in health outcomes for men along gender, sometimes referred to as the cost of masculinity [8]. Patriarchy and *machismo* culture are, in the end, detrimental to both men and women. Men have a lower life expectancy than women, with the gap between male and female life expectancy widening [9]. Men are less likely to utilize health services than women, and the gender norm of traditional masculinity stigmatizes against self-care, unemployment, and other health-relevant aspects of life in a male-specific way [8,9,10]. Despite these documented disparities, the topic of men’s health has not been a significant focus of research and policy efforts, especially in Latin America. A new report from the Pan-American Health Organization (PAHO) emphasizes the importance of studying masculinities related to health outcomes given the impact of gender norms in *machismo* culture in Latin America [10]. The report indicates that the hegemonic forms of masculinity should be considered a risk factor for health based on men’s limited socialization, identity-formation, and cultural participation, especially in a retirement stage of life where a man no longer can be defined by his profession [10]. Women tend to have more robust social networks than men. It seems that “women’s tendency to put a premium on their social connections is one of the main reasons they live longer” [1]. While the economic and patrimonial aspects of gender inequity heavily impact women, the socio-emotional implications of traditional gender roles are detrimental to men. Men are frequently treated as mere obstacles in gender research and policy, which has become synonymous with “women”. True promotion of health equity requires treating men as both diverse and as allies in building healthier communities [10].

As a middle-income country, Ecuador is undertaking both a demographic and epidemiological transition [11]. The life expectancy in Ecuador is 77.7 years for both sexes, 80.5 for women and 75.1 for men [12]. The health system requires restructuring to address an increase in the elderly population (above 65 years of age) and the rising incidence of non-communicable disease. While care for the elderly through nuclear and extended families is culturally understood as ubiquitous across all Ecuadorian populations, research from the Ecuadorian Sierran highlands indicates that social networks may not be as integrated conventionally perceived [13]. Only after social movements throughout the twentieth century, culminating in the new Constitution of 2008, did the state formally recognize the full multicultural and multinational identity of its citizenry [14,15]. The historical marginalization of indigenous populations continues to impact today, especially in regions that are more rural and/or historically indigenous, where social isolation occurs at higher rates [11,13]. The majority of the Ecuadorian population self-defines as *mestizo* [11], a mix between *indigena* (indigenous) and European (White) descent, which results from a complicated history wherein *mestizos* were afforded full rights of citizenship (including access to land, health and education) whereas indigenous populations were not [14]. Other ethnic demographics in the Sierra region include afro-Ecuadorian or *negro* (Black), *montubio* (coastal *campesinos*, or farmworkers), and *mulatto* (historically understood as a mix between Black and Caucasian/*Mestizo* Ecuadorians) [11,14].

The Ecuadorian Ministry of Health relies on a framework for integrated health, the *Modelo de Atención Integral de Salud* (MAIS), that finds basis in upholding the rights and entitlements of all citizens. MAIS addresses social determinants of health while promoting “family, community, and intercultural health” [16]. Cumbaya Valley locates itself in the Sierran highlands, east of the capital city of Quito, and forms one of two agrarian valleys that border the urban locale. Within hours of the capital city with proper transport, it has historically been home to a marginalized indigenous population and still contains many remote areas. Publicly funded community health centers, one pertaining to each town in the Valley, execute the *MAIS* framework at the community level in partnership with *Universidad de San Francisco de Quito* (USFQ) Medical School. Through the *Programa de Atención Integral de la Salud* (Integrative Health Care Program), USFQ worked with local community health centers in 2015 to cultivate equitable partnerships that benefit the communities in the Cumbaya Valley [17]. The *Clubes de Adultos Mayores* (Elderly Clubs), free of charge and growing in popularity, have been established at each clinical site. The weekly Elderly Clubs allow participants to engage socially with members of their local community and medically with health professionals and medical students. The programming acts as a sustainable form of community engagement in each town while also introducing medical students to community-based learning that encourages social integration as an aspect of well-being.

As Elderly Clubs take root in a clinical environment, community providers see the weekly social gathering as a form of physical health management (i.e., blood pressure monitoring, nutrition promotion, physical exercise promotion, etc.). Often overlooked is the importance of mere human congregation:

Surprisingly, face to face social capital in a neighborhood can predict who lives and who dies even more powerfully than whether the area is rich or poor. In 2003, when several Harvard epidemiologists put nearly 350 neighborhoods under the microscope, they discovered the social capital – as measured by reciprocity, trust, and civic participation – was linked to a community´s death rates. The higher the levels of social capital, the lower its mortality rates [18].

Even the health providers who promote the Elderly Clubs may not fully understand the implications of such groups. The clinic itself can become a place of equitable social integration that strengthens community health and well-being.

Participation rates and activities vary across the location. One constant in the various clubs, however, is a large gender disparity in participation. The gender distribution of the Elderly Clubs is roughly 80% women and only 20% men. This study investigates the quality and extent of men’s social integration in the Cumbaya Valley to better understand why men are less likely to attend the community health center clubs and develop ideas for increasing male participation.

## Methods

### Study Population

This observational study was completed in conjunction with the Latitude-0 Ecuador Research Initiative of USFQ and collaboration with the USFQ Medical School. Data collection took place from April through July 2019. Study participants were 100 men 40 years of age or older from the following five demographically similar towns in the Valley of Tumbaco: El Quinche, Lumbisi, Pifo, Puembo, and Tumbaco. Participants were recruited from the five health centers from each of these communities through a community-based partnership previously established between the USFQ Medical School and the Ministry of Health. Initially, only four sites were consulted for the study population. When access to the Tumbaco clinical site became difficult due to administrative difficulties within the Ministry of Health, researchers transitioned to the neighboring community of Lumbisi to collect the remaining surveys. Based on clinical records of the older people who attend the El Quinche and Pifo clinics, only 0.4% and 1% identify as indigenous, respectively. Recruitment occurred through convenience sampling at the health centers, during at-home medical visits, and through local community groups. All participants spoke Spanish. Surveys were administered through interviews between participants and research assistants affiliated with USFQ. All study materials and procedures were approved by the Comité de Ética de Investigación en Seres Humanos at USFQ (IRB), and all materials containing sensitive personal identity information were guarded at USFQ.

### Study Instrument and Procedure

General demographics were collected for all study participants following the standardized structure of previously utilized surveys developed at USFQ for the Cumbaya Valley’s elderly population Table 1. The composite survey (Sup1SIEM.pdf) compiled two previously validated surveys from the Social Participation and the Health and Well-Being of Canadian Senior Citizens (SPHWB) survey [19] as well as the Medical Outcomes Study (MOS) Social Support Survey [20]. The MOS data allows insight into the subjective experiences of loneliness, isolation, community engagement, and affective love experienced by each study participant. The SPHWB provides a baseline of understanding the types and frequency of social interaction in which participants engage. The qualitative component of the study included conversing with men who did not participate in social activity clubs. They shared both the causes for their lack of participation in these groups and their ideas on how to increase participation. Regardless of age or participation, all participants were welcome to provide suggestions for new programming at the Elderly Clubs.

**Table 1:** Demographic characteristics of study participants.


**Age (years, mean ±SD)**	**67.5 ±13.1**		
**Education (%)**		**Housing (%)**	
Illiterate	18	Rented	23
Incomplete primary	22	Owned	57
Primary	29	Belongs to child	9
Incomplete secondary	9	Other	11
Secondary	11	**Live alone? (%)**	
Incomplete University	1	Yes	20
University	10	No	80
Race (%) **Self-Identified**		**Work (%)**	
Indigenous	16	Never worked	3
Black/Afro Ecuadorian	3	Retired	29
Mestizo	71	Unemployed	22
Mulato	4	Working	46
White	0		
Montubio	5		
Other	3		
No Response	3		

Efficacy in translation to local, culturally-appropriate language was verified by native, Spanish-speaking staff at USFQ. Verification included a two-step process in which the survey was first reviewed and edited by USFQ Medical staff and then further tested by administering three mock surveys/interviews with male students of USFQ medical school. Data collection, assisted via the KoboToolBox data analysis platform, resulted in 100 valid surveys.

### Statistical Analysis

Upon achieving 100 total surveys, data collection was considered sufficient and exhausted. Data were analyzed using Stata/SE 15.1 software, and statistical significance was set at an alpha value of 0.05. Means and standard deviations for continuous variables, and percentages for nominal variables were determined. Geometric means of each social participation score were compared to the means of each subcategory using paired t-tests. A linear regression model was used to assess the association between social participation scores and selected demographic characteristics.

## Results

### Quantitative Results

**Figure 1:**
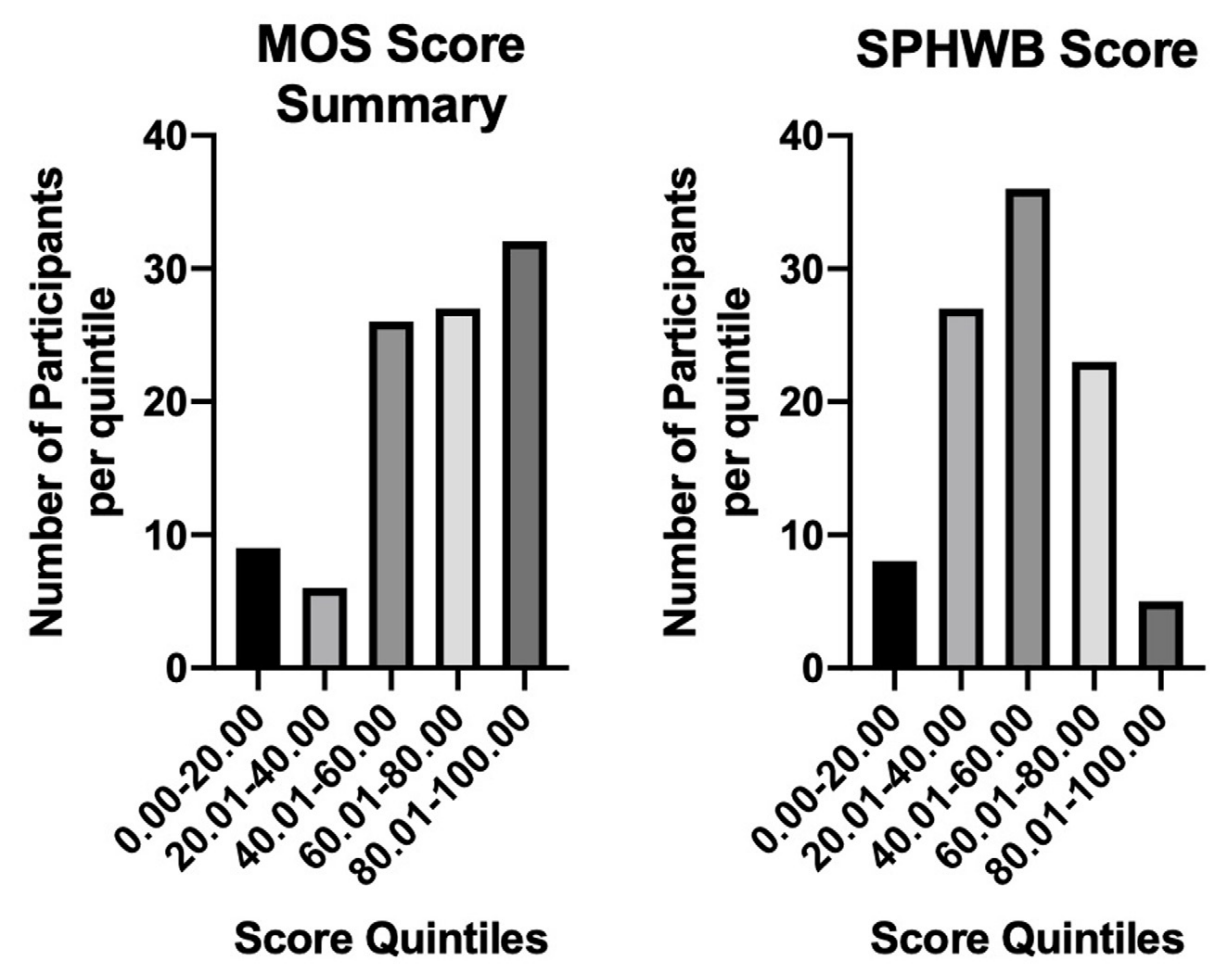
Social Score: MOS and SPHWB.

In responses to the Medical Outcomes Survey, which indicates the subjective experience of social support, overall scores largely fell in the top 3 quintiles. 15% of participants produced low, answering “never” or “rarely” to most questions posed about social support. The SPHWB, which examines the types of people with whom participants interact and the types of activities they undertake, has more varied results. The majority of patients reside in the middle three quintiles (Figure 1).

**Figure 2:**
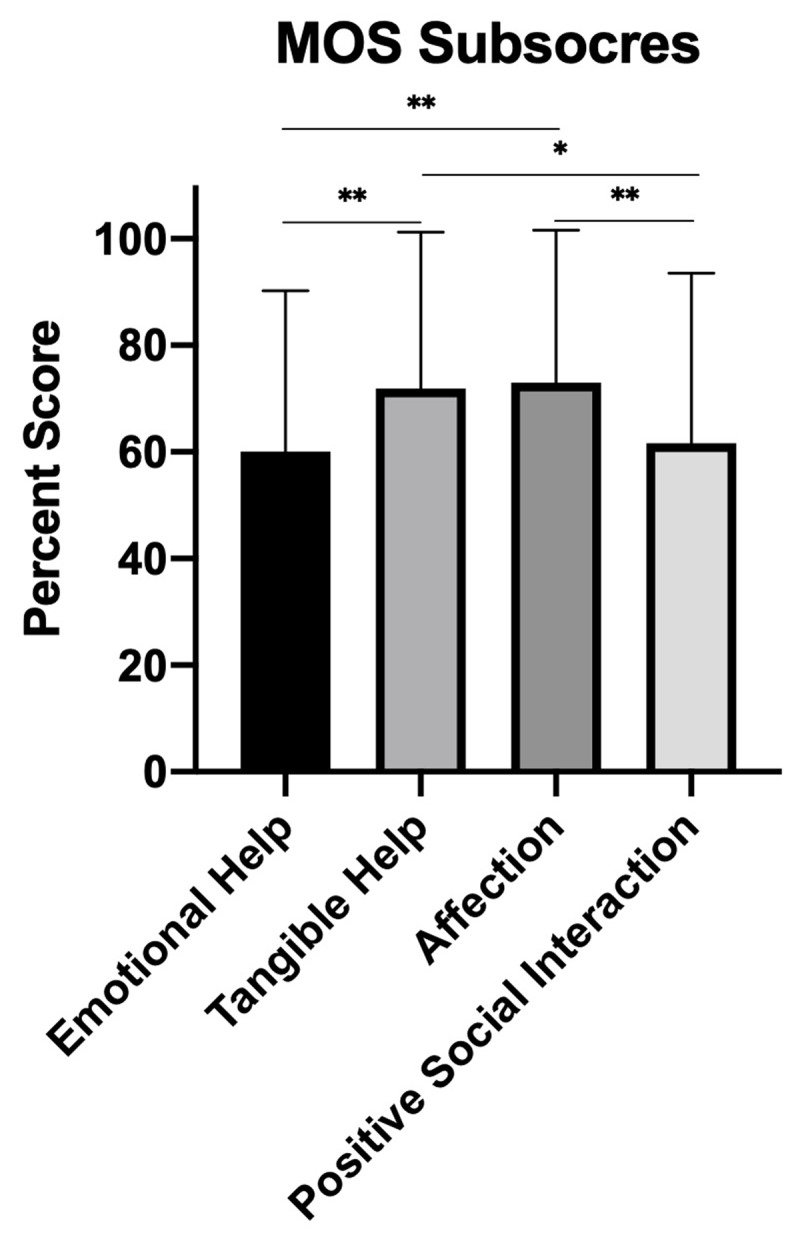
Male Social Participation by MOS subscore.

The emotional and social subscores are not statistically significantly different from each other (p = 0.7156). The subscores for affectionate and tangible help are not different from each other (p = 0.7917). However, the emotional subscore is statistically significantly less than the affectionate subscore (p = 0.0022) and the tangible subscore (p = 0.0054). Similarly, the positive social participation subscore is statistically significantly less than the affectionate subscore (p = 0.0088) and the tangible subscore (p = 0.0189). Overall, participants demonstrate higher access to tangible and affectionate support than emotional and positive social participation Figure 2.

**Figure 3:**
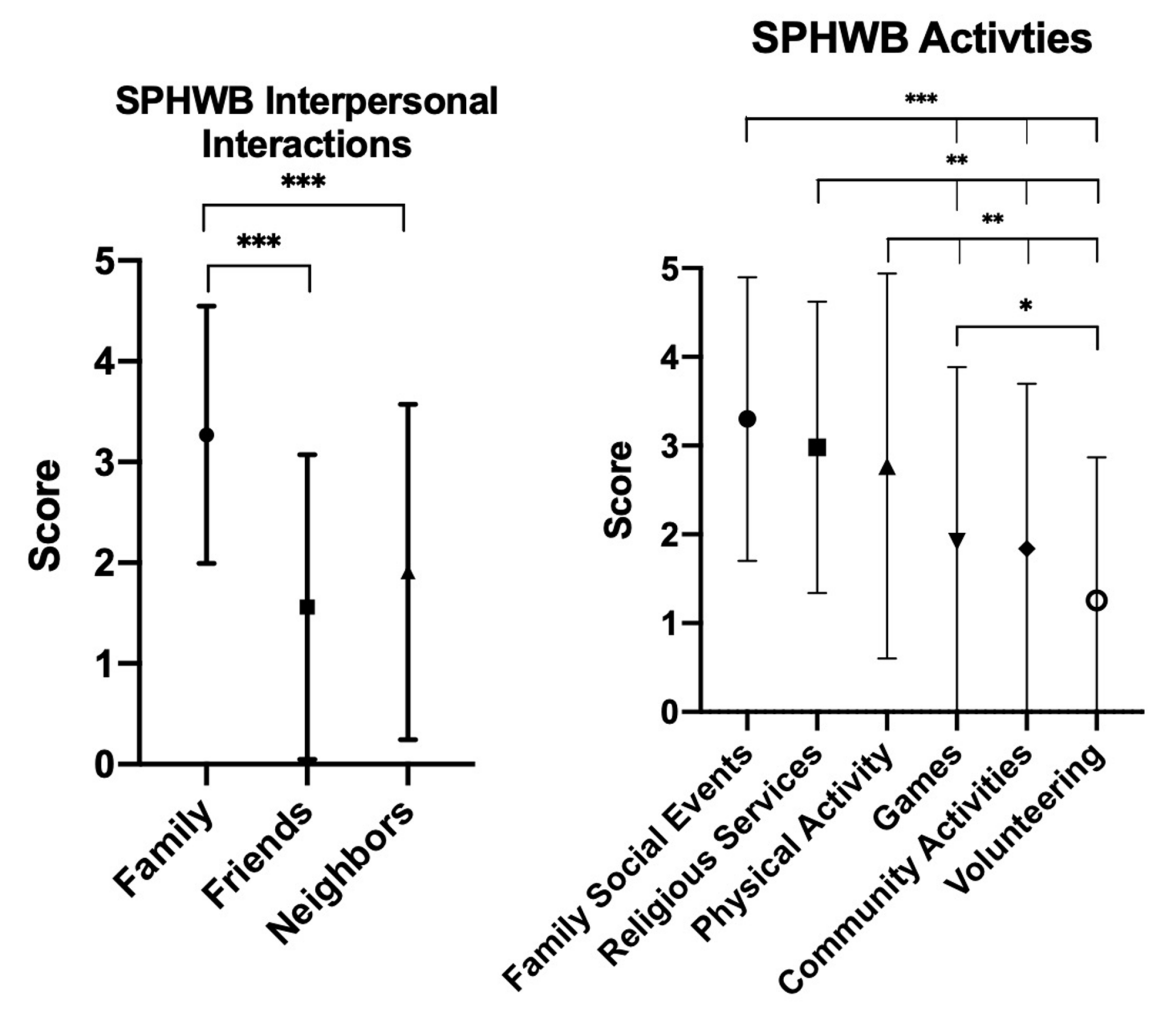
Means of SPHWB subcategories.

In considering the SPHWB questions (Table 2), participants were more likely to frequently participate in social activities with their family than with neighbors or friends Figure 3.

**Table 2:** Summary of SPHWB responses.

	Activities						Interpersonal Interaction		

	Familial Outings	Religious Services	Physical Activity	Games	Neighborhood Activities	Volunteering	Time w/ family	Time w/ friends	Time w/ neighbors

Mean (±SD)	3.300 (1.600)	2.980 (1.641)	2.770 (2.169)	1.919 (1.967)	1.838 (1.861)	1.255 (1.278)	3.270 (1.278)	1.560 (1.513)	1.910 (1.664)

Age is negatively related to the MOS score until age 68.96, at which point each additional year is related to an increase in the score Table 3. With increased education, there is an increased social score. There were no associations between other demographic components (race, housing, employment) and overall social integration scores.

**Table 3:** Regression results for social participation scores and selected demographic characteristics.

	Age	Age Squared	Education

MOS	–4.364 (1.860)*	0.031 (0.014)*	5.070 (1.347)*
SPHWB	–0.882 (1.409)	0.005 (0.011)	3.370 (1.047)*

**Figure 4:**
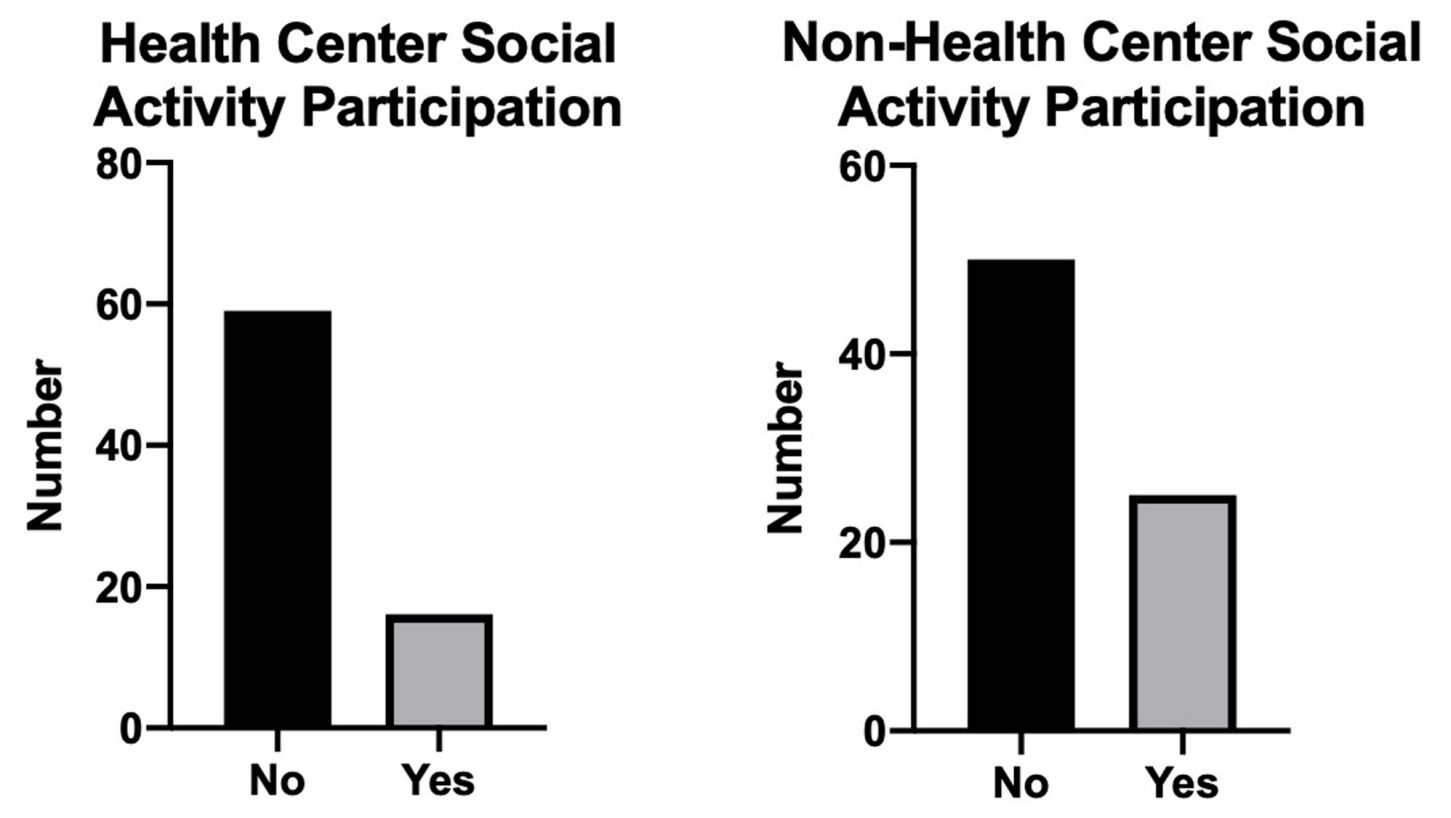
Structured elderly social participation.

The majority of study participants over the age of 60 did not participate in an Elderly Club through the Health Center or the municipality (Figure 4).

### Qualitative Results

The authors determined two categories from qualitative results: barriers to access community health programming and community-suggested solutions for increasing male participation in Elderly Club activities.

### Barriers to Health Center Programs

Many study participants cited specific reasons for not participating in the Elderly Clubs sponsored by the clinics.

**Figure 5:**
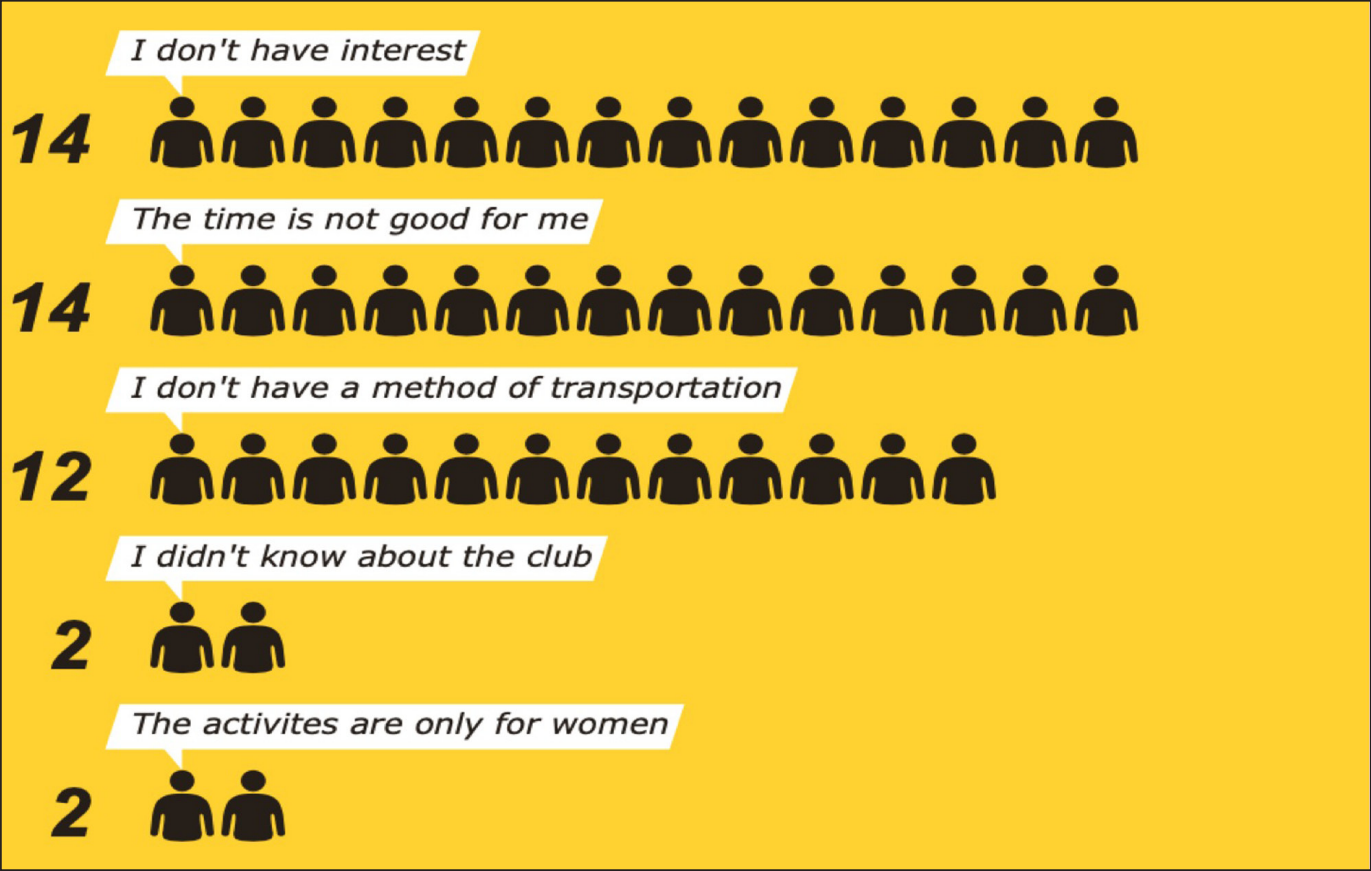
Barriers to Elderly Club Attendance.

A total of 37 respondents provided answers to these preset responses. Some respondents gave multiple answers (Figure 5).

In terms of time conflicts, work was cited five times. Transportation issues, six of which were specifically cited as health-related, were mentioned as another main barrier to attendance. A few interviewees agreed to become members of the club after the interview concluded, demonstrating that established interest exists, but the communication and coordination of the clubs’ times and activities might be lacking.

One participant describes why his recent medical history influenced his participation:

“I cannot mobilize myself easily. I have had operations on my knee and hip.”

Another 78-year-old man echoes a similar sentiment:

“Before we used to come, but due to the pain [we no longer attend]”.

Although participants cite barriers to participating in the club, the barriers do not necessarily indicate disinterest. A 74-year-old man explains:

“I cannot leave my house much because of the state of my health. I only have support in the morning most days because my children work, and I have a niece who also works.”

Scheduling compounds accessibility issues for some, especially with those who have caregivers and cannot leave the house by themselves. One participant’s daughter, who acts as his caregiver, explained:

“All of the programs are always on a fixed schedule; it is difficult to bring [my elderly relatives]”.

Attendance for those with limited physical mobility is highly unlikely given the current scheduling and social (including at home) support systems.

The roles of needing care, as well as being a caregiver, can limit a person’s ability to engage in social life beyond the family. Some older men asserted that they did not attend the club because they had to fulfill caretaking duties for fellow family members:

“I like physical activities, I cannot leave my wife for whom it is difficult to leave the house and has the risk of falling, but yes, I like to go out and do activities when I can.”

While the barriers listed above are related to non-gender specific dilemmas, many gender-specific implications did arise. One participant explained why he does not attend the Elderly Club:

“Because my wife does not want to go.”

He explained that while he did enjoy the activities of the club – and did want to attend – he would only attend alongside his wife. While interested, he does not feel empowered to participate without the social support of his wife. In this situation, a peculiar and important phenomenon appears: The participant demonstrated a social dependence on his wife.

Further responses highlight gendered attitudes towards social life in general and the Elderly Clubs in particular:

“The man defines himself through his work and family.”

“Men do not socialize; maybe they don’t want to go with their spouse.”

“[Men] don’t want to come, sometimes I am ashamed [to attend the club]”.

Men cite their gender, gendered social expectations, and social stigma as barriers to club attendance. Even one man who attends experiences shame from his community for partaking in the activities. Men perceive the Elderly Clubs as an unwelcoming environment for men:

“They don’t appreciate us, men, very much… not all men are bad, and we care about the rest of society and our families.”

Addressing the barriers to access requires solutions that are supported by community members and address both the gender specific and gender non-specific barriers to Elderly Club attendance.

### Community Suggested Solutions

Male interviewees provided several unique suggestions for increasing male participation. Some men also expressed interest in becoming involved directly with the programming initiatives and working in an organizing role.

**Figure 6:**
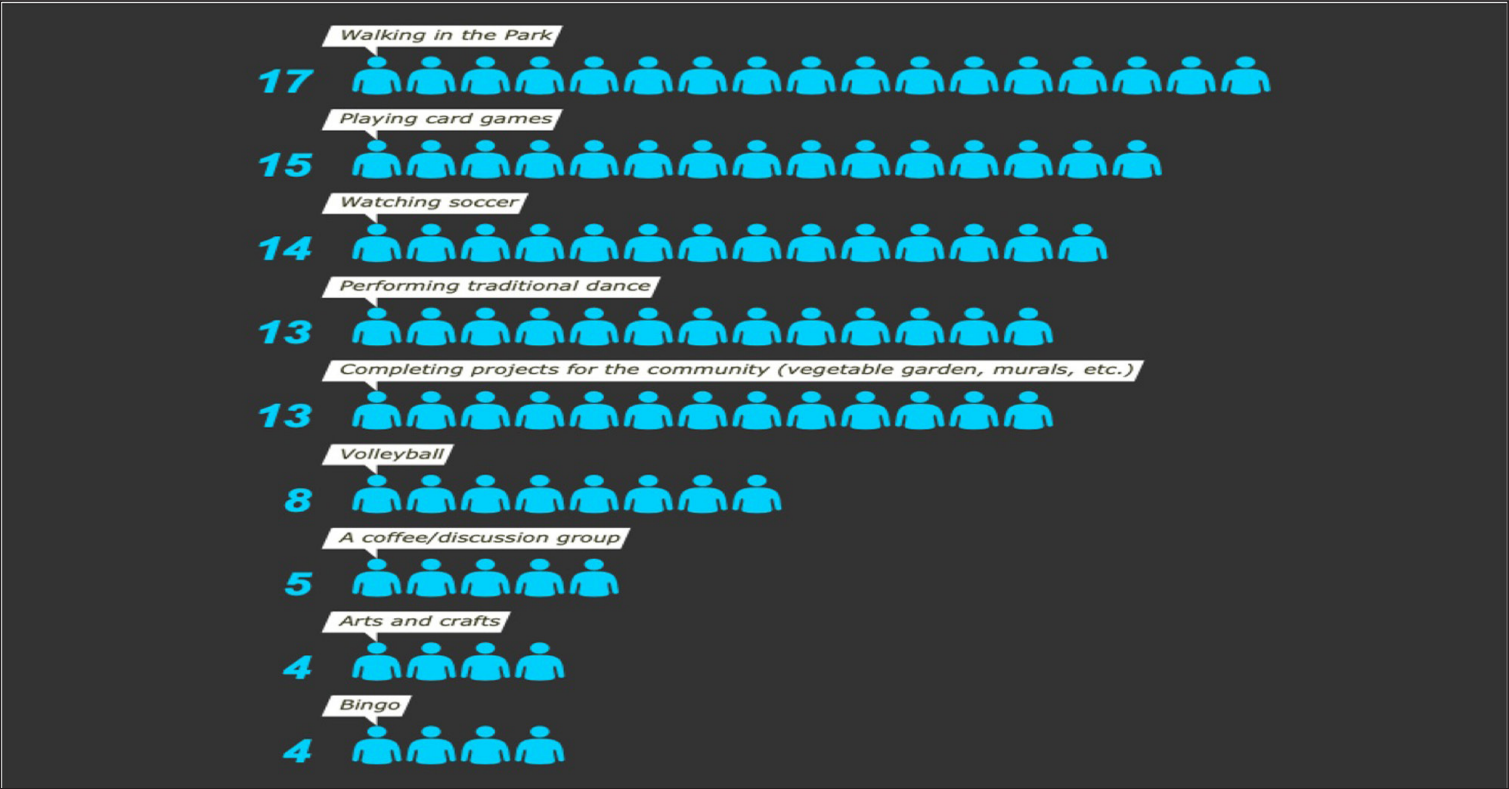
Community Suggested Responses.

A total of 34 respondents provided answers to these preset responses. Some respondents gave multiple answers (Figure 6).

Common responses included various types of exercise, conversation/socializing, or community work. Recommendations ranged in feasibility, depending on the age and integral health of participants. In the interviews, many of the men cited that the club should be more inclusive towards older men specifically:

“I would like to see more ‘strength-based’ activities.”

“When it is your own space, you can do more things. People dance in their homes, play national songs… [then] maybe I’ll come to the club.”

“Men are more closed off [than women], there are people that live far away within Pifo, it’s such a big place…[I’d suggest] volleyball, sports, beers, and food.”

“We need to change the name; there is a stigma related to age.”

Specific pleas for “more masculine” activities as well as the indications that it currently does not feel like one’s own space demonstrates an implicit, even sometimes explicit, understanding that not all are welcome. The name of the club, while using the more formalized and respectful Spanish term to reference elderly persons, stigmatizes some who think the term *adulto mayor* categorizes them in a negative light.

Many study participants supported the idea of starting a community garden from the health clinic:

“There are different needs – emotional, physical. [The club must be] more than something superficial, especially in physical and mental terms. [Men] have worked for the most part out in the fields on the land, and men continue an agricultural way of life [as they age]”.

In addition to the cultivation of fruits and vegetables, the provision of snacks or meals were listed (noted in five interviews) to be the main attraction for men to participate.

A further suggestion was made by one man in Pifo who belonged to the local Jehovah’s Witness parish. He suggested that door-to-door invites, similar to the style of his church, might positively increase the number of participants in the community clubs. He further invited the interviewer to attend the church to promote the club and complete more interviews with men.

## Discussion

This is the first study to evaluate social participation in adult men in Latin America, and the study brings to light a need for more attention in this regard. Strengths included quick and efficient engagement with the community, with simultaneous data collection as well as the promotion of currently available resources. Many men of the Cumbaya Valley did not have high social integration scores and could benefit from increased social activity. Men were more likely to have access to tangible and affective support than emotional aid or positive social interaction (Figure 2). This difference may arise because men interact with people outside of the family with a significantly lower frequency (Figure 3). Men in the Cumbaya Valley would benefit from stronger, more integrated social networks.

More information must be consulted to understand the impact of age on social integration, given its unique trend. As people age, they may realize an increased desire to experience connection and stronger social networks. The tangible realities of retirement and increased physical dependency may also allow for greater social connection. An alternative explanation might posit that people who have more robust social networks and social support tend to live longer; those living longer would be expected to have a higher social score as a result of the protective nature of their lifestyle.

Education was positively associated with social integration scores. We hypothesize that, just as education is associated with better overall health, higher education levels may promote better social wellbeing, through either helping to establish stronger and larger social networks or increasing self-efficacy. Age was positively associated with social scores until the age of 69, at which point it became negatively associated. We believe this is a result of shrinking social networks that come with older age. While education and age did trend with higher levels of participation, employment, as well as housing, did not show to have any significant impact on social engagement.

The health center groups are largely attended by women, and most men that participate are the husbands of women in the clubs. Men generally feel uncomfortable, especially on their own. People are more willing to join if they have friends or acquaintances who already participate and assume an already normalized role (i.e., husband of a woman who has female friends in the group) within the community. Many men may experience social dependency on their female partners.

Many of the study participants held strong attitudes towards men’s role in social life, even though questions never specifically intended to acquire older men’s views on gender and sexuality. The study participants expressed normative sentiments regarding masculinity. Many believed that men should not have social commitments beyond those of work and family. The types of social activities promoted at the health clinic appear to be culturally understood as feminine. As expressed in the PAHO Masculinities Report, a *machismo* culture is circumscribing social participation for men [9]. Requests for more traditionally masculine activities should be taken seriously, and a wider conversation should begin regarding limiting gender roles that diminish health equity.

Gardening was strongly supported by male community members and has shown efficacy when piloted in Pifo. Especially given the large population of elderly persons that worked in agriculture before retirement, this activity welcomes both men and women equally. The leaders of the garden initiative in Pifo, who planted most plants and tended to it daily, consist of both men and women who work together. The garden, which lies in the backyard of the clinic, increases the number of informal interactions throughout the week between clinical staff and the community members and addresses one current barrier to accessing Elderly Clubs: a strict schedule.

Communities of faith could become another manner to integrate elderly community members who already have a platform for community-based engagement. Such programming already exists in places such as El Quinche, where the local medical staff visit the Reina de El Quinche parish to complete medical check-ups and initiate social engagement for the elderly each week.

The study design faced several limitations. Convenience sampling, while generally a reliable method for pilot testing, limited the representative nature of the results. Further, while the interview/questionnaire allowed for qualitative analysis, a structure was not in place to gather the more detailed stories of participants nor formally collect suggestions for the clubs at each clinic. The study design focused on frequency scales, but the actual number of social connections was not tracked. For example, a person with only one social connection may respond that he “always has someone to give him a hug,” but that access could qualitatively differ from a person who is constantly surrounded by several doting relatives, friends, and acquaintances. Local health providers were not formally consulted to understand their perspective on male participation in Elderly Clubs.

## Conclusions and Suggestions

Research conducted with communities in the Cumbaya Valley demonstrates the need for more social spaces that integrate male community members. Clinical staff and university collaborators may conduct further research through means such as focus groups to understand social barriers faced by men and to implement more inclusive programming. Understanding an individual’s social web – rather than a mere subjective perspective on socialization – would provide more a firm understanding of the communities’ needs. This becomes especially important, considering that overlapping social relationships are protective of health [1]. Given the trend of age with social integration levels, it may be best to target younger populations before reaching an age of higher social abandonment. Since older populations often may require increased caretaking and transportation support, programming could be designed to decentralize the gathering of the groups or facilitate community-based resources dedicated to transportation and/or accompaniment. As the groups are largely attended by women, they may act as safe social support for women. However, older men currently feel excluded by the structure of programming. New approaches must maintain the current communal nature of the Elderly Clubs while addressing disparities in participation. Further research should continue to address gender-based drivers of access inequity at the local community health level. Addressing male-specific needs may include an expansion of the existing Elderly Clubs or the formation of new groups.

A final citation represents the sentiment described by many of the older men interviewed:

“When I arrive [to the Elderly Club], it is important that people hug me, that they listen to me. All of us need to be heard. [You should] take care of your dad, listen to the things from his whole life.”

The local community clinic can be a space in which the Village Effect is reproduced. For some in the Cumbaya Valley, *Los Clubes de Adulto Mayores* already lift the capacity for such social integration, a capacity that must be revitalized in a safe and equitable manner as and when COVID-19 protocols allow. More research and engagement, especially focused on male individuals who face gender-specific stigmatization, will ensure that a positive and supportive community is facilitated for all.

## Additional File

The additional file for this article can be found as follows:

10.5334/aogh.3020.s1 Appendix A. Cuestionario para Participación Social en Hombres.
