# Supplementary material for: Social Integration and Community Health Participation of Elderly Men in Peri-Urban Ecuador
Source: Ann Glob Health. 2020 Oct 26;86(1):138. doi: 10.5334/aogh.3020 (PMC7597577; doi:10.5334/aogh.3020)
Supplement: Appendix A. — Cuestionario para Participación Social en Hombres. [file agh-86-1-3020-s1.pdf]

Cuestionario para Participación Social en Hombres

Criterio de inclusión:

- Hombres mayor que 40 años que viven en Pifo, El Quinche, Tumbaco o Puembo

1. Demografía
    a. Fecha de la entrevista __-__-____
    b. En el caso de que la entrevista la realice un/a cuidador/a
    c. Sexo del/a cuidador/a
        i. Hombre 0
        ii. Mujer 1
        iii. Otrx 2
        iv. No es aplicable 99
    d. Edad del/a cuidadora ____
    e. Relación con el entrevistado
        i. Cónyuge 0
        ii. Yerno/nuera 1
        iii. Hermano/a 2
        iv. Amigo/a 3
        v. Cuñado 4
        vi. Cuidador/a 5
        vii. Hijo/a 6
        viii. Otro/a 7
        ix. Ns/nc 99
    f. ¿Podría darme su (número) de cédula? _____________-__
    g. ¿Cuál es su nombre? (entrevistado sujeto de estudio)
    h. ¿Puede decirme su edad? _______
    i. Fecha de nacimiento __-__-____
    j. Dirección, Barrio
    k. Teléfono
    l. Teléfono contacto (Tipo y de quien)
    m. ¿Cuáles son sus estudios?
        i. Analfabeto 0
        ii. Primaria Incompleta 1
        iii. Primaria 2
        iv. Secundaria Incompleta 3
        v. Secundaria 4
        vi. Universidad Incompleta 5
        vii. Universidad 6

n. ¿Usted se considera?

| | | |
|---|---|---|
| i. | Indígena | 0 |
| ii. | Afro ecuatoriano (negro) | 1 |
| iii. | Mestizo | 2 |
| iv. | Mulato | 3 |
| v. | Blanco | 4 |
| vi. | Montubio | 5 |
| vii. | Otro | 6 |
| viii. | Ns/nc | 99 |

o. ¿El lugar donde vive usted…?

| | | |
|---|---|---|
| i. | Es arrendada/alquilada | 0 |
| ii. | Es su propia | 1 |
| iii. | Pertenece a su hijo/a | 2 |
| iv. | Otra | 3 |

p. ¿Vive usted solo?

| | | |
|---|---|---|
| i. | Sí | 0 |
| ii. | No | 1 |

q. Sí si, ¿Con quien vive? ____________________

2. Trabajo

a. ¿Usted trabaja actualmente?

| | |
|---|---|
| No, nunca trabajé | 0 |
| No, jubilado | 1 |
| No, estoy desempleado | 2 |
| Sí | 3 |

SPHWB Survey

3. ¿Con qué frecuencia hace usted las siguientes actividades?

a. Sale de la casa para hacer actividades sociales con familia o amigos

| | |
|---|---|
| Nunca | 0 |
| Una vez al año | 1 |
| Cada 2-3 meses | 2 |
| Una vez al mes | 3 |
| Una vez a la semana | 4 |
| Casi Todos los días | 5 |

b. Va a misa o servicios religiosos

| | |
|---|---|
| Nunca | 0 |
| Una vez al año | 1 |
| Cada 2-3 meses | 2 |
| Una vez al mes | 3 |
| Una vez a la semana | 4 |
| Casi Todos los días | 5 |

c. Hace actividad física/deporte con otras personas

| | |
|---|---|
| Nunca | 0 |
| Una vez al año | 1 |
| Cada 2-3 meses | 2 |
| Una vez al mes | 3 |
| Una vez a la semana | 4 |
| Casi Todos los días | 5 |

d. Otras actividades con amigos o familia como juegos de carta, Bingo, pasatiempos

| | |
|---|---|
| Nunca | 0 |
| Una vez al año | 1 |
| Cada 2-3 meses | 2 |
| Una vez al mes | 3 |
| Una vez a la semana | 4 |
| Casi Todos los días | 5 |

e. Actividades con la comunidad o el barrio

| | |
|---|---|
| Nunca | 0 |
| Una vez al año | 1 |
| Cada 2-3 meses | 2 |
| Una vez al mes | 3 |
| Una vez a la semana | 4 |
| Casi Todos los días | 5 |

f. Voluntariado o trabajo de apoyo a la sociedad/comunidad

| | |
|---|---|
| Nunca | 0 |
| Una vez al año | 1 |
| Cada 2-3 meses | 2 |
| Una vez al mes | 3 |
| Una vez a la semana | 4 |
| Casi Todos los días | 5 |

4. ¿Con qué frecuencia interactúa usted con los siguientes grupos de personas?
   a. Con familia?

| | |
|---|---|
| Rara vez | 0 |
| Una vez al mes | 1 |
| Una vez a la semana | 2 |
| Casi todos los días | 3 |
| Todos los días | 4 |

   b. Con amigos?

| | |
|---|---|
| Rara vez | 0 |
| Una vez al mes | 1 |
| Una vez a la semana | 2 |
| Casi todos los días | 3 |
| Todos los días | 4 |

c. Con vecinos?

| | |
|---|---|
| Rara vez | 0 |
| Una vez al mes | 1 |
| Una vez a la semana | 2 |
| Casi todos los días | 3 |
| Todos los días | 4 |

Medical Outcomes Study (MOS) Social Support Survey

5. ¿Con qué frecuencia tiene acceso a estos tipos de apoyo? **Apoyo emocional o informativo**.
   a. Apoyo de una persona en quien puede confiar si necesita hablar

| | |
|---|---|
| Nunca | 0 |
| Pocas veces | 1 |
| A veces | 2 |
| Muchas veces | 3 |
| Siempre | 4 |

b. Apoyo de alguien que le ayuda a comprender una situación o problema

| | |
|---|---|
| Nunca | 0 |
| Pocas veces | 1 |
| A veces | 2 |
| Muchas veces | 3 |
| Siempre | 4 |

c. Alguien que le puede aconsejar en una crisis emocional, económica, etc.:

| | |
|---|---|
| Nunca | 0 |
| Pocas veces | 1 |
| A veces | 2 |
| Muchas veces | 3 |
| Siempre | 4 |

d. Una persona en quien puede confiar sus problemas:

| | |
|---|---|
| Nunca | 0 |

| | |
|---|---|
| Pocas veces | 1 |
| A veces | 2 |
| Muchas veces | 3 |
| Siempre | 4 |

e. Alguien que le aconseja cuando lo necesita:

| | |
|---|---|
| Nunca | 0 |
| Pocas veces | 1 |
| A veces | 2 |
| Muchas veces | 3 |
| Siempre | 4 |

f. Alguien en quien puede compartir sus preocupaciones y miedos:

| | |
|---|---|
| Nunca | 0 |
| Pocas veces | 1 |
| A veces | 2 |
| Muchas veces | 3 |
| Siempre | 4 |

g. Alguien que le puede aconsejar en un problema personal:

| | |
|---|---|
| Nunca | 0 |
| Pocas veces | 1 |
| A veces | 2 |
| Muchas veces | 3 |
| Siempre | 4 |

h. Alguien que entiende sus problemas:

| | |
|---|---|
| Nunca | 0 |
| Pocas veces | 1 |
| A veces | 2 |
| Muchas veces | 3 |
| Siempre | 4 |

6. ¿Con qué frecuencia tiene acceso a estos tipos de apoyo? **Apoyo tangible**

a. Alguien que le podría ayudar si usted no pudiera salir de su cama:

| | |
|---|---|
| Nunca | 0 |
| Pocas veces | 1 |
| A veces | 2 |
| Muchas veces | 3 |
| Siempre | 4 |

b. Alguien que le puede llevar al médico en caso de estar enfermo:

| | |
|---|---|
| Nunca | 0 |
| Pocas veces | 1 |
| A veces | 2 |
| Muchas veces | 3 |
| Siempre | 4 |

c. Alguien que le podría preparar la comida si usted no pudiera hacerlo:

| | |
|---|---|
| Nunca | 0 |
| Pocas veces | 1 |
| A veces | 2 |
| Muchas veces | 3 |
| Siempre | 4 |

d. Alguien que le podría ayudar con las tareas diarias, si estuviera enfermo

| | |
|---|---|
| Nunca | 0 |
| Pocas veces | 1 |
| A veces | 2 |
| Muchas veces | 3 |
| Siempre | 4 |

e.

7. Con qué frecuencia tienes acceso a estos tipos de apoyo? **Apoyo de cariño**

a. Alguien que le muestra amor y cariño:

| | |
|---|---|
| Nunca | 0 |
| Pocas veces | 1 |
| A veces | 2 |
| Muchas veces | 3 |
| Siempre | 4 |

b. Alguien que le hace sentir amado y valorado:

| | |
|---|---|
| Nunca | 0 |
| Pocas veces | 1 |
| A veces | 2 |
| Muchas veces | 3 |
| Siempre | 4 |

c. Alguien que le abraza:

| | |
|---|---|
| Nunca | 0 |
| Pocas veces | 1 |
| A veces | 2 |

| | |
|---|---|
| Muchas veces | 3 |
| Siempre | 4 |

8. ¿Con qué frecuencia tiene acceso a estos tipos de apoyo? **Interacción Social Positiva**
   a. Alguien con quien usted puede pasar un buen rato, un momento feliz, agradable, bonito:

| | |
|---|---|
| Nunca | 0 |
| Pocas veces | 1 |
| A veces | 2 |
| Muchas veces | 3 |
| Siempre | 4 |

   b. Alguien con quien usted se puede reunir para relajarse:

| | |
|---|---|
| Nunca | 0 |
| Pocas veces | 1 |
| A veces | 2 |
| Muchas veces | 3 |
| Siempre | 4 |

   c. Alguien con quien puede divertirse:

| | |
|---|---|
| Nunca | 0 |
| Pocas veces | 1 |
| A veces | 2 |
| Muchas veces | |
| Siempre | 4 |

**(para hombres >60 años)**

9. Usted participa en el club de adultos mayores en el centro de salud?

| | |
|---|---|
| No | 0 |
| Sí | 1 |

10. Usted participa en algún otro actividad social en su barrio (60 y piquito, etc.)?

| | |
|---|---|
| No | 0 |
| Sí | 1 |

(Si no):

   a. Por qué no participa?

| | |
|---|---|
| No me interesan las actividades | 0 |
| La hora no es buena para mi | 1 |
| No tengo medio de transporte | 2 |
| No sabía del club | 3 |

Actividades son solo para mujeres 4
…
Otra razón (especificar): ________________

b. ¿Usted iría si hiciéramos otras actividades?
¿Como cuales?

| | |
|---|---|
| Póker/40/juego de cartas | 0 |
| Mirar futbol | 1 |
| Bingo | 2 |
| Baile | 3 |
| Club de café y discusión | 4 |
| Caminar en el parque | 5 |
| Obras para la comunidad (como jardinería, ir a guardería, etc.) | 6 |
| Hacer/usar cometas | 7 |
| Hacer carritos (coches) de madera o resorteras | 8 |
| Jugar volley | 9 |
| Realizar crucigramas | 10 |

…

c. (Aplica a todos:) Otra ideas para actividades o clubes sociales (especificar):

___________________

Protocolos Utilizados:

Social Participation and the Health and well-being of Canadian Senior Citizens
https://www.researchgate.net/publication/232607486_Social_participation_and_the_health_and_well-being_of_Canadian_seniors#:~:text=Social%20participation%20and%20the%20health%20and%20well%2Dbeing%20of%20Canadian%20seniors,-Article%20(PDF%20Available&text=Background%3A%20Social%20participation%20has%20been,well%2Dbeing%20in%20older%20adults.&text=Results%3A%20An%20estimated%2080%25%20of,at%20least%20one%20social%20activity.

Medical Outcomes Study (MOS) Social Support Survey
https://www.rand.org/health/surveys_tools/mos/social-support/survey-instrument.html
